# Supplementary material for: Isolation and Quantification of L-Tryptophan from Protaetia brevitarsis seulensis Larvae as a Marker for the Quality Control of an Edible Insect Extract
Source: Insects. 2025 Aug 29;16(9):905. doi: 10.3390/insects16090905 (PMC12471237; doi:10.3390/insects16090905)
Supplement: Supplementary file 1 [file insects-16-00905-s001.zip › insects-3793203-supplementary.pdf]

Supplementary Data

Isolation and Quantification of L-Tryptophan from *Protaetia brevitarsis seulensis* Larvae as a Marker for Quality Control of Edible Insect Extract

Hye Jin Yang and Wei Li*

Korean Medicine (KM) Application Center, Korea Institute of Oriental Medicine, Daegu 41062, Republic of Korea; hjyang @kiom.re.kr

* Correspondence: liwei1986@kiom.re.kr

Contents

Figure S1. HPLC-DAD chromatograms of the reference L-tryptophan standard, the isolated compound, and the three batches of the 70% EtOH extract of *P. brevitarsis* larvae (PBE)

Figure S2. HPLC-DAD chromatograms of PBE and its fractions (PBE-H, PBE-E, PBE-B, and PBE-W) derived from *P. brevitarsis* larvae

Figure S3. Overlaid HPLC-DAD chromatograms of the reference L-tryptophan standard, the isolated compound, the 70% EtOH extract of *P. brevitarsis* larvae (PBE), and the *n*-BuOH fraction (PBE-B), with an enlarged view of the highlighted region

Figure S4. HR-ESI-MS spectrum of the major peak in the PBE-B fraction

Figure S5. ^{1}H NMR spectrum of the isolated compound (400 MHz, D_2O)

Figure S6. ^{1}H NMR spectrum of the isolated compound (400 MHz, D_2O)

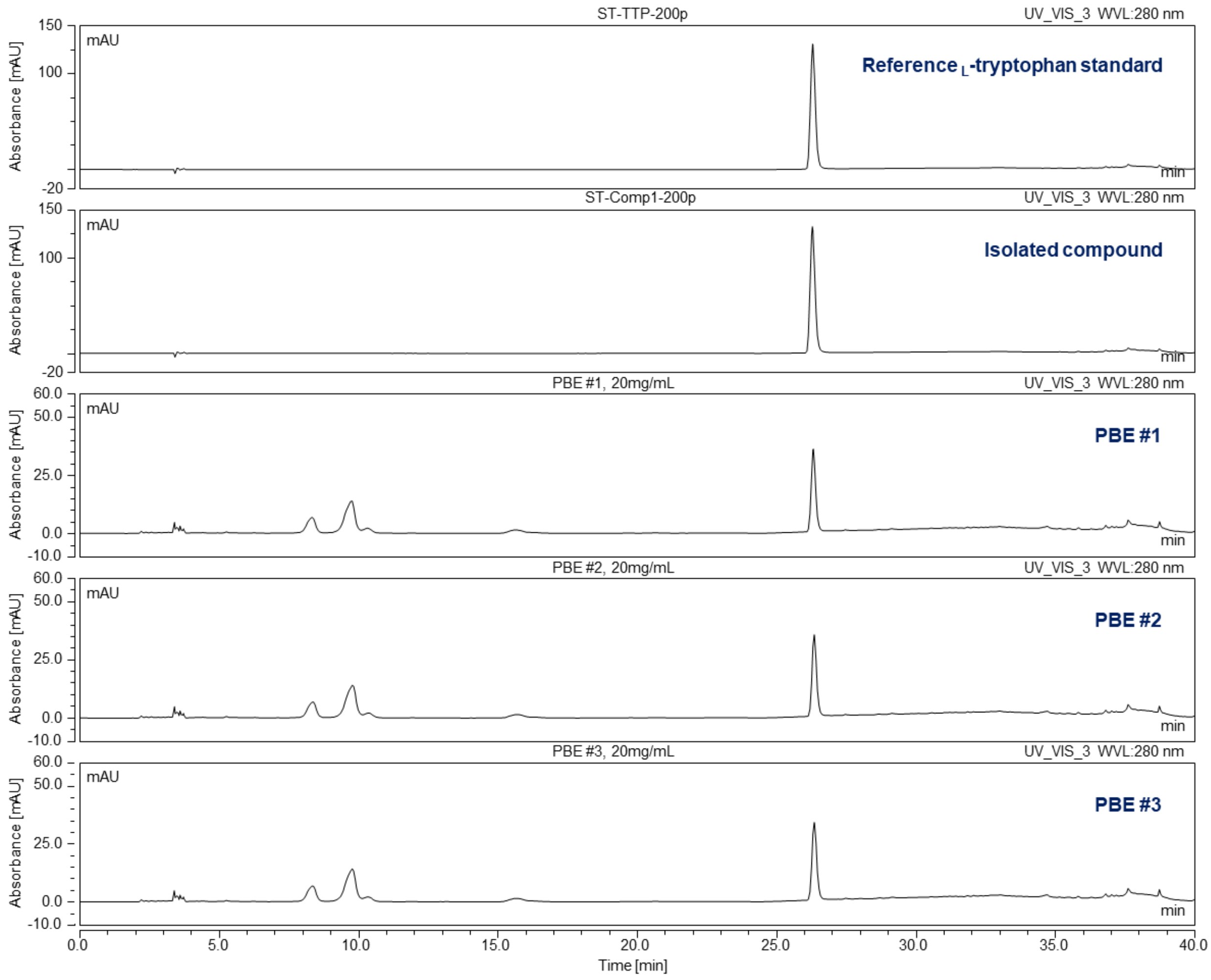

Figure S1. HPLC-DAD chromatograms of the reference L-tryptophan standard, the isolated compound, and the three batches of the 70% EtOH extract of *P. brevitarsis* larvae (PBE)

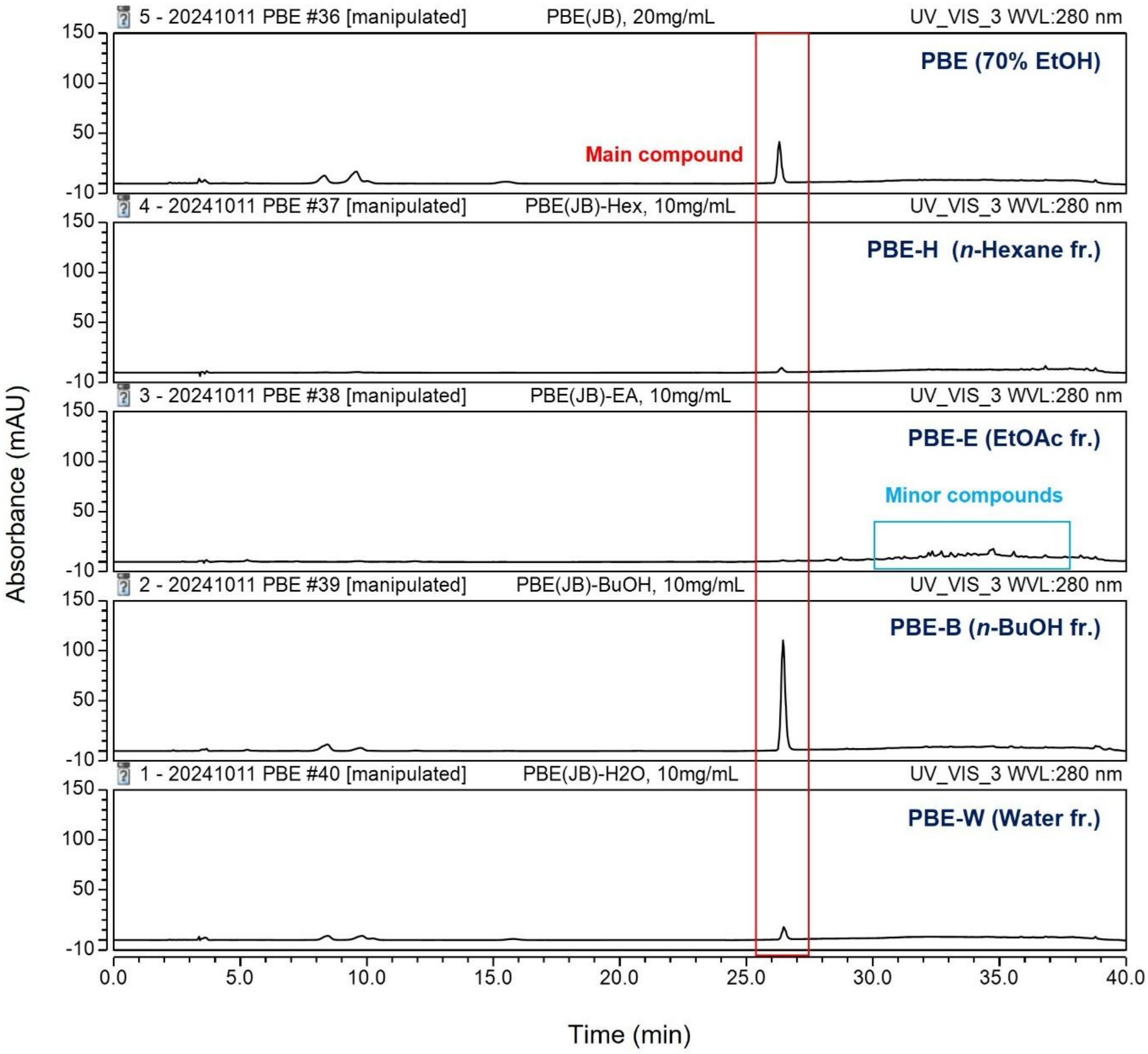

Figure S2. HPLC-DAD chromatograms of PBE and its fractions (PBE-H, PBE-E, PBE-B, and PBE-W) derived from *P. brevitarsis* larvae

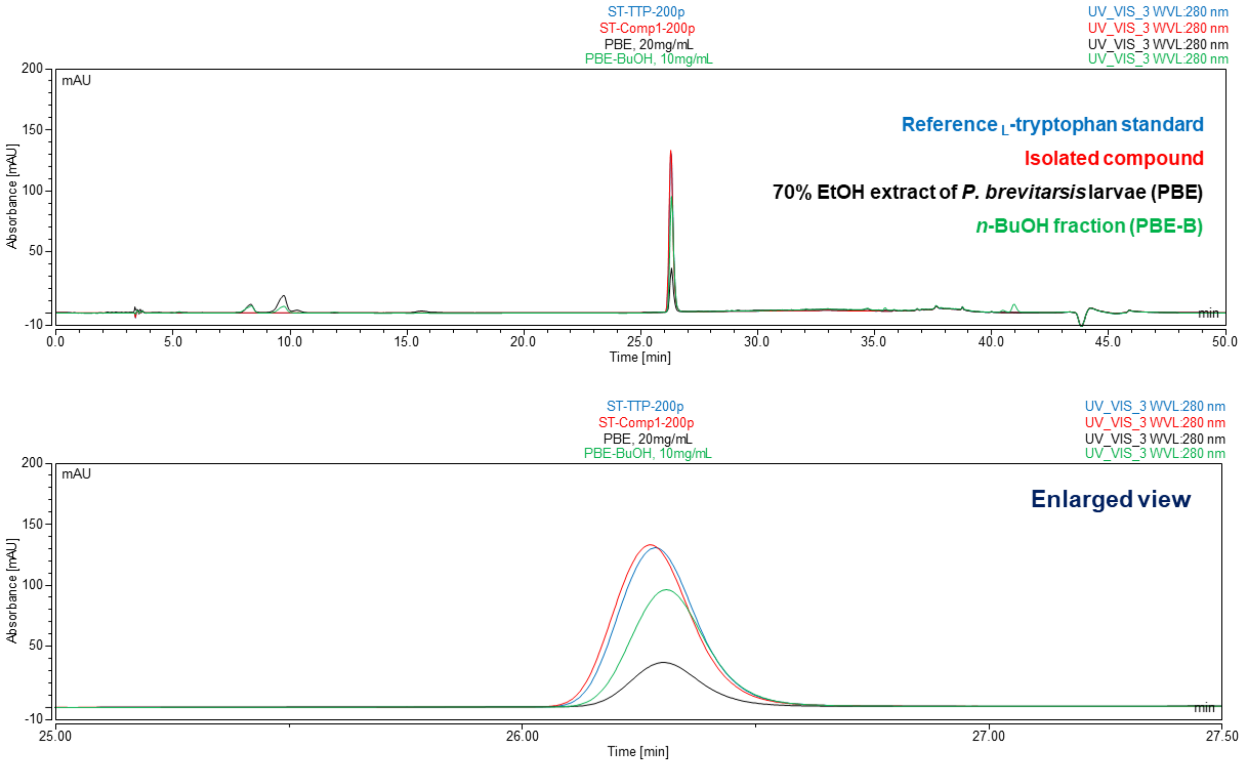

Figure S3. Overlaid HPLC-DAD chromatograms of the reference L-tryptophan standard, the isolated compound, the 70% EtOH extract of *P. brevitarsis* larvae (PBE), and the *n*-BuOH fraction (PBE-B), with an enlarged view of the highlighted region

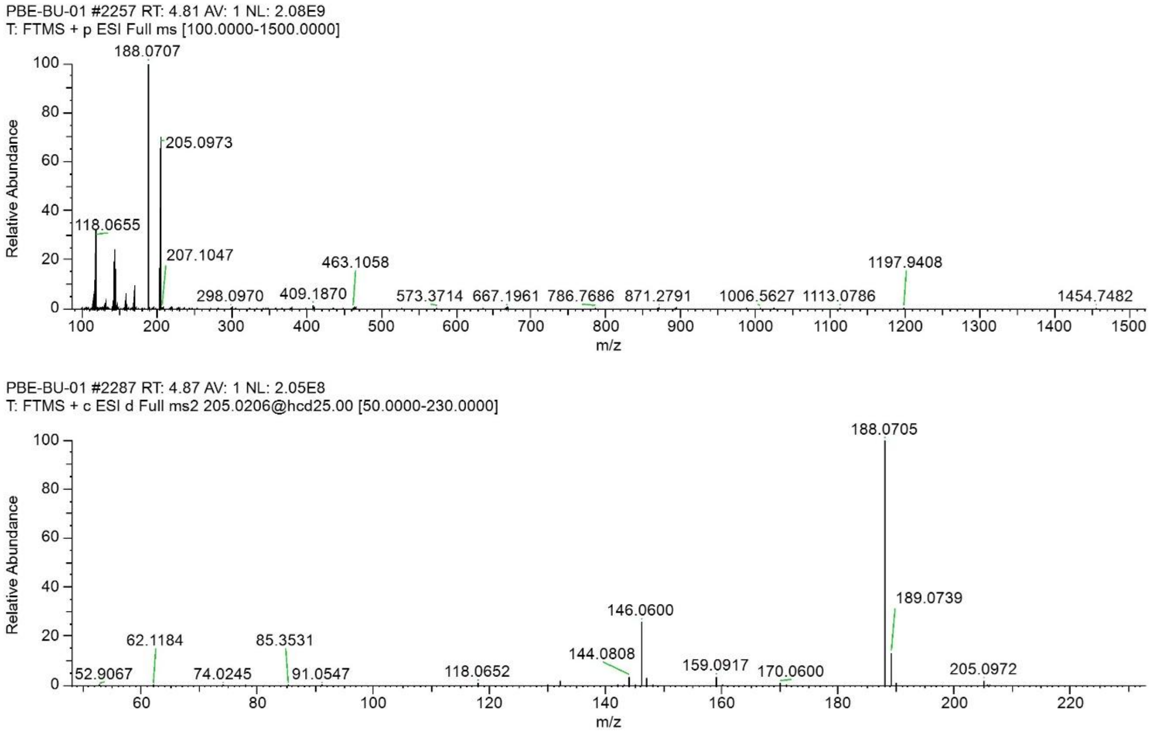

Figure S4. HR-ESI-MS spectrum of the major peak in the PBE-B fraction

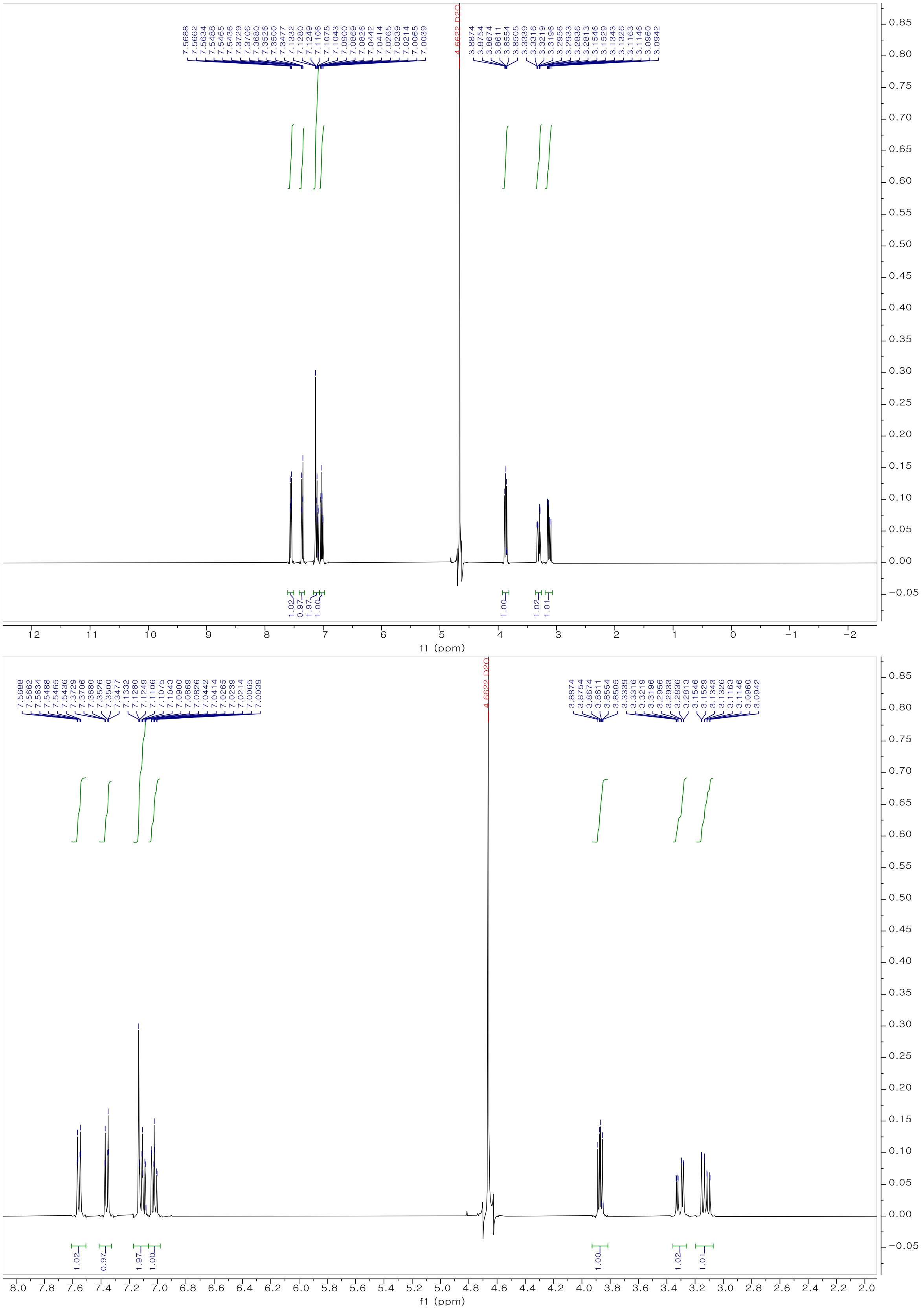

Figure S5. ^1H NMR spectrum of the isolated compound (400 MHz, D_2O)

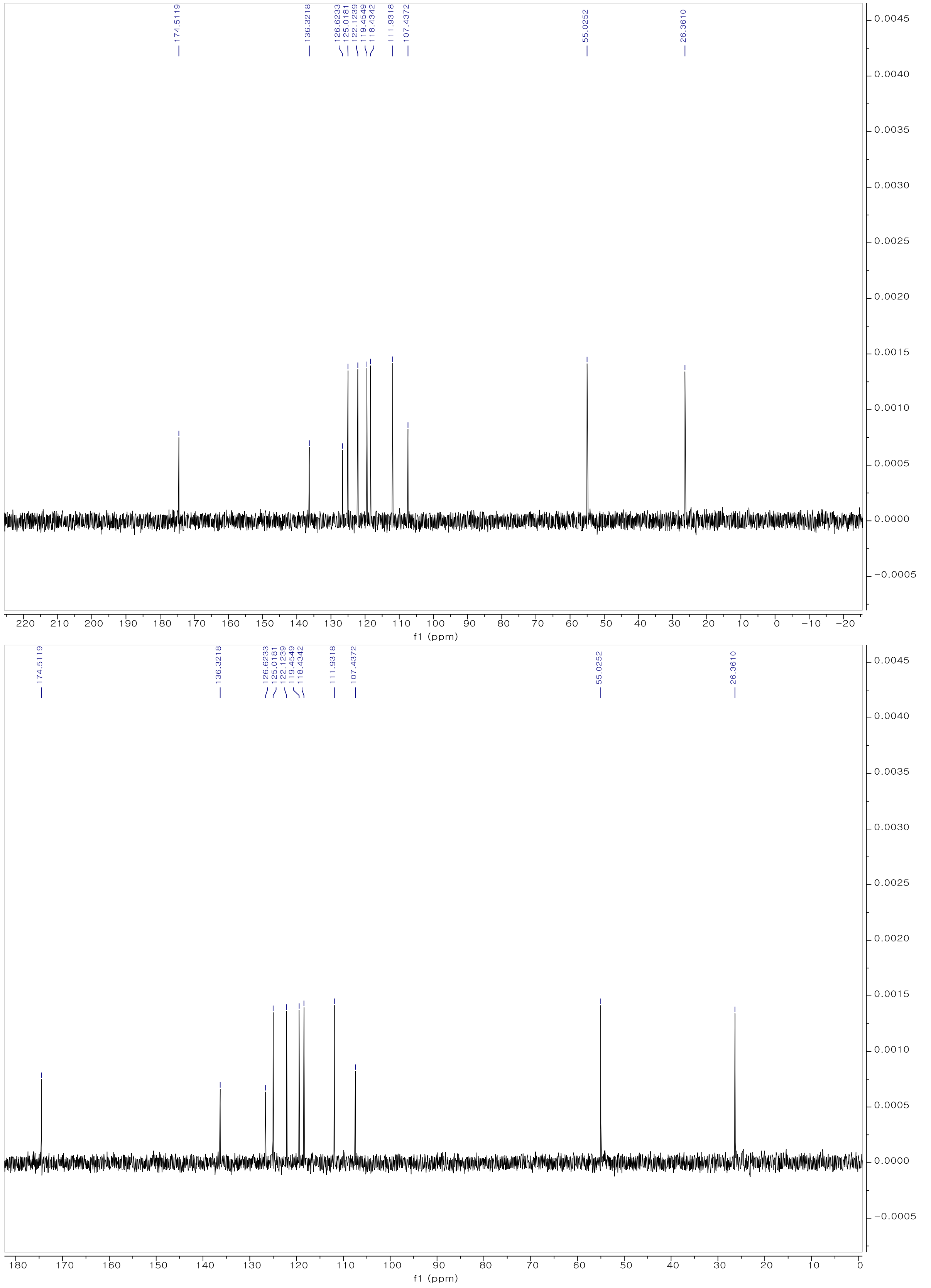

Figure S6. ^{13}C NMR spectrum of the isolated compound (100 MHz, D_2O)
